# Investigation of Secondary Metabolites and Their Bioactive Potential in Various *Iris* Species and Cultivars Grown under Different Cultivation Conditions

**DOI:** 10.1021/acsomega.5c06354

**Published:** 2025-10-20

**Authors:** Tereza Jaegerova, Jitka Viktorova, Marie Zlechovcova, Miroslav Vosatka, Petr Kastanek, Jana Hajslova

**Affiliations:** † Department of Food Analysis and Nutrition, Faculty of Food and Biochemical Technology, 52735 University of Chemistry and Technology Prague, Technicka 5, 166 28 Prague, Czech Republic; ‡ Department of Biochemistry and Microbiology, Faculty of Food and Biochemical Technology, University of Chemistry and Technology Prague, Technicka 5, 166 28 Prague, Czech Republic; § Department of Biotechnology, Faculty of Food and Biochemical Technology, University of Chemistry and Technology Prague, Technicka 5, 166 28 Prague, Czech Republic; ∥ EcoFuel Laboratories s.r.o., Ocelarska 9, 190 00 Prague 9, Czech Republic

## Abstract

This study analyzed the bioactive secondary metabolites in leaves and roots with rhizomes of various *Iris* species and cultivars (subgenus *Iris* and *Limniris*) grown under different conditions. Plants were cultivated in aeroponics and hydroponics and treated with a feather hydrolysate as an alternative nutrient source or arbuscular mycorrhizal fungi (AMF) to promote metabolite production. Phytochemical profiling was performed by using UHPLC-HRMS/MS, aided by in silico fragmentation for compound identification. Chemometric analyses revealed differences in the metabolic profiles between plant parts, including upregulation of phenolic acids, steroids, and *C*-glycosyl xanthones in the leaves and of stilbenes, triterpenoids, and benzophenones in the roots with rhizomes. Variations between the subgenera *Iris* and *Limniris* revealed possible chemotaxonomic markers, while cultivars of the same species showed a close similarity. Hydroponics resulted in upregulation of 232 metabolites, including flavone and isoflavonoid glycosides, compared to 42 upregulated metabolites in aeroponics, like xanthones and benzofurans. Neither feather hydrolysate nor AMF significantly altered the profile of detected metabolites. The correlation of the phytochemical profiles with biochemical assays of the *Iris* extracts indicated metabolites with cytotoxic, antimicrobial, and anti-inflammatory potential, including both known bioactive compounds (e.g., iridin, phalerin, or *p*-coumaric acid) and compounds with no published bioactivity data (e.g., irisjaponin A).

## Graphical abstract

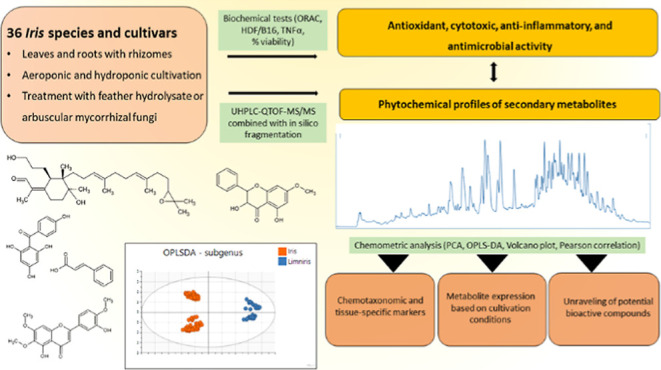

## 1 Introduction

The *Iris* plants are cultivated both for ornamental purposes and industrial applications, with one of the most renowned uses being the production of orris butter, an esteemed substance highly sought after in the fragrance and cosmetics industries.^1,2^ In recent years, irises have received considerable attention for their array of biological and therapeutic properties, including antimicrobial, antiviral, antioxidant, anti-inflammatory, antidiabetic, antiosteoporotic, hepatoprotective, phytoestrogenic, anticholinesterase, antihyperglycemic, antihyperlipidemic, anticarcinogenic, antiplasmodial, and molluscicidal activities.^2–6^ These diverse attributes render them invaluable resources for medicine as well as various industrial sectors, focusing on diet, health, and beauty. Additionally, *Iris* extracts find application in the food sector as additives, contributing color and flavor.^1^ At present, the species of industrial significance, include *Iris pallida*, *Iris germanica*, and *Iris Florentina*. Nevertheless, the *Iris* genus, the largest within the Iridaceae family, comprises over 300 species,^7^ each with a potentially distinct metabolic profile, bioactive properties, and industrial potential. Yet, the phytochemical profile and bioactivity of many of these species and cultivars remain underexplored or unexplored.

A review of the existing literature revealed nearly 300 reported secondary metabolites across various *Iris* species, many of which are responsible for the biological activities observed in the extracts. These metabolites encompass a wide range of compound groups, including flavonoids, isoflavonoids, phenolic acids, phenols, stilbenes, xanthones, quinones, sterols, steroids, and terpenoids, some of which have been attributed a chemotaxonomic value.^4,8–17^ Environmental factors, such as soil composition, nutrient availability, pH, temperature, or humidity, have been shown to influence the production, accumulation, and distribution of secondary metabolites in plants.^18–20^ For instance, phosphorus and potassium levels in the soil, along with sunlight exposure, significantly affect the accumulation of phenolic compounds in various *Iris* species.^19^ These external factors are difficult to control in conventional field cultivation, resulting in variable concentrations of bioactive metabolites in the same plants cultivated across different regions and times.^20^

Alternative cultivation methods, such as hydroponics and aeroponics, offer more precise control over environmental variables, allowing for the potential standardization of the bioactive metabolite content. In hydroponics, nutrients are delivered in liquid nutrient-rich media rather than through traditional soil,^20^ while aeroponics involves the aerosolized application of nutrients to plant roots and stems suspended in the air.^21^ Recently, a trend has emerged to use industrial byproducts as a source of these essential nutrients. An example is chicken feathers, which pose environmentally problematic waste that is challenging to dispose of, but, in the hydrolyzed form, could serve as a low-cost growth medium.^22^ While these alternative cultivation approaches undisputably benefit from the sustainable water usage without the need for soil and space saving through vertical cultivation, contradictory results have been reported for different plants on the ability to increase the production of biomass and bioactive metabolites.^23,24^ To date, to the best of our knowledge, no studies have compared the metabolic profiles of *Iris* species cultivated in aeroponic and hydroponic systems.

Another factor influencing plant metabolism is the treatment with arbuscular mycorrhizal fungi (AMF), which has been shown to enhance the nutrient uptake, reduce phytotoxicity from heavy metals, increase stress tolerance, and protect against pathogens.^25–29^ In *I. germanica*, AMF inoculation has been linked to the increased accumulation of primary metabolites, such as starch and myristic acid.^30^ However, to the best of our knowledge, the influence of AMF on secondary metabolite production in *Iris* species remains unexplored.

Despite extensive research on the chemical composition and biological activity of extracts prepared from various *Iris* species (e.g., ref 2, 4, 5, 8, 10, 15 and 31–42 ), many species and cultivars have until now received little to no attention. Furthermore, many analytical studies tend to focus on a limited range of phytochemicals, failing to capture the full metabolic diversity of these plants and possibly overlooking metabolites of significant biological and industrial value. Reliable compound identification is crucial for enabling further biochemical investigation and ensuring safe and proper industrial applications. Conventional LC-UV/DAD methods generally provide a low identification confidence for compounds in crude plant extracts, unless analytical reference standards are used. Tandem high-resolution mass spectrometry (HRMS/MS) is a technique that produces characteristic fragmentation (MS/MS) spectra of compounds used to deduce the molecular structure. Particularly when coupled to separation techniques, such as liquid chromatography (LC), HRMS/MS offers a more reliable compound identification. However, the fragmentation spectra generated by LC-HRMS/MS can be rather difficult to compare when obtained under different instrumental conditions. Moreover, the labor-intensive process of manually reviewing and comparing MS/MS spectra to external spectral libraries, together with the lack of MS/MS spectra and the absence of reference standards for many plant metabolites, makes identification challenging. Under these circumstances, in silico fragmentation is a tool that utilizes advanced computational algorithms to predict molecular structures from fragmentation data, enabling the high-throughput screening of hundreds of metabolites with an improved identification confidence.^43^

The presented bioprospective study explores the phytochemical profiles of a unique collection of various *Iris* species and cultivars and provides insights into the effect of different cultivation conditions to foster industrial potential. The implemented innovative UHPLC-HRMS/MS aided by in silico fragmentation allowed the screening of 288 *Iris* secondary metabolites from an in-house database, representing various chemical classes, including flavonoids and isoflavonoids, phenolic acids, xanthones, terpenoids, steroids, and others. In addition, bioactivity tests were performed on the *Iris* extracts, and the results were correlated to the metabolic profiles to identify potential bioactive compounds. Key aims were to (i) compare the phytochemical profiles of leaves and roots with rhizomes of various *Iris* species and cultivars from subgenus *Iris* and *Limniris* and identify chemotaxonomic markers; (ii) investigate the effect of aeroponic and hydroponic cultivation systems on the detected metabolites; (iii) assess the effect of AMF inoculation and treatment with a feather hydrolysate during growth on the phytochemical profile; and (iv) identify compounds correlated with antimicrobial, anti-inflammatory, antioxidant, and cytotoxic activity.

## 2 Results and Discussion

This study employed UHPLC-HRMS/MS to analyze 149 samples of 37 different *Iris* plants cultivated under four distinct conditions (aeroponics, hydroponics, treatment with a feather hydrolysate, and treatment with AMF). A detailed overview of the samples is provided in Supporting Information, S1. The analysis provided a broad data matrix of a total of 747 detected compounds, matching the exact mass and isotopic profile of known *Iris* secondary metabolites from our in-house database (Supporting Information, S2) and passing through the MSC filter where applicable, as described in Section 4.6. UHPLC-HRMS/MS data processing and compound identification. The number of detected compounds surpassed the 288 metabolites included in the in-house database, primarily owing to the recurrent occurrence of isomeric peaks, which inherently complicates unambiguous compound identification, as explained in Section 4.6. The detected compounds represented predominantly (iso)­flavonoids (460), terpenoids (78), and phenols (74), with smaller numbers of phenolic acids (41), xanthones (29), quinones (25), steroids (15), stilbenes (8), and other unclassified compounds (17). As expected and consistent with previous reports, (iso)­flavonoids were the most abundant secondary metabolites in *Iris*.^40,44^ The lowest number of targeted metabolites (103) was found in the leaves of the hydroponically grown, AMF-treated *Iris lactea* (sample 114), while the highest number (282) was detected in the roots with rhizomes of the hydroponically grown, AMF-treated *Iris squalens* (sample 133). The phytochemical profiles of these two samples are illustrated in Figure 1. The signals of the detected metabolites across the different samples and their tentative identifications based on the in-house database are provided in Supporting Information, S3.

**Figure 1:**
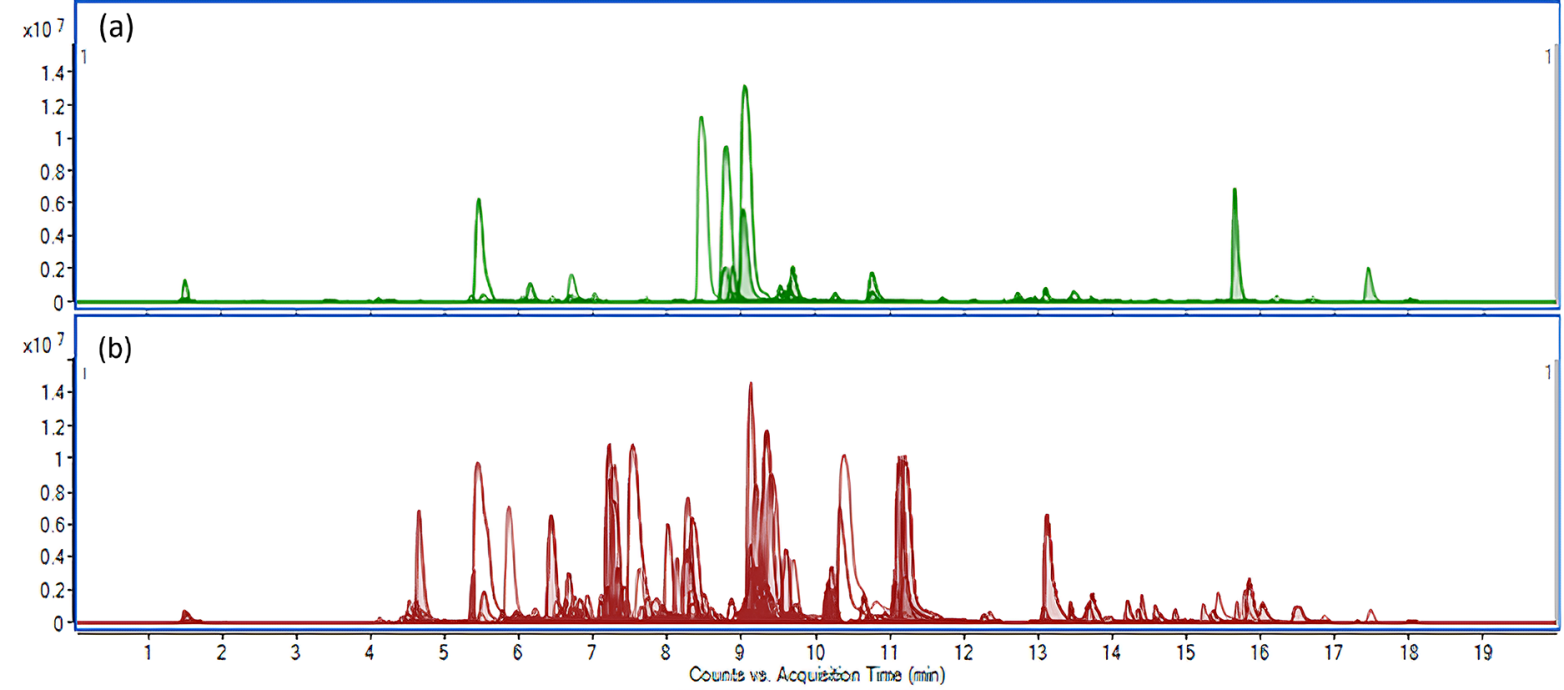
Phytochemical profile of *Iris* extracts with the lowest and highest number of detected metabolites. Overlaid extracted ion chromatogram (EIC) of compounds detected in positive and negative ionization modes in the extracts of (a) leaves of the hydroponically grown AMF-treated *Iris lactea* (sample 114, 103 metabolites) and (b) roots with rhizomes of the hydroponically grown, AMF-treated *I. squalens* (sample 133, 282 metabolites) based on the in-house metabolite database.

Given the high complexity of the data due to the multiple experimental variables, namely, *Iris* subgenus, species, cultivar, plant part, cultivation method, and plant treatment during growth, principal component analysis (PCA) was performed on the complete data set to identify general trends (Figure 2). The first two principal components of the PCA explained 61.6% of the variance, and one of the key findings was the clear separation of samples based on plant part, leaves versus roots with rhizomes. Further distinctions were observed among the leaf samples based on the cultivation method, aeroponic versus hydroponic. Among the roots with rhizomes, *Iris versicolor* grown in aeroponics did not cluster with the other aeroponic samples, suggesting that other factors may be driving its separation. This led to the identification of an additional grouping, expressed especially within the roots with rhizomes, based on subgenus variation, *Iris* versus *Limniris*. The separation of *I. versicolor* roots with rhizomes from other aeroponic samples likely reflects its classification under the subgenus *Limniris*, which was the only such representative among the aeroponic samples. This observation hints at the presence of chemotaxonomically significant metabolites in *I. versicolor*. With respect to plant treatments during growth - feather hydrolysate in aeroponics and AMF in hydroponics - the PCA revealed no substantial alterations in metabolic profiles, as treated and untreated samples clustered closely together. The implications of PCA were further investigated and are discussed within the following sections of this chapter.

**Figure 2:**
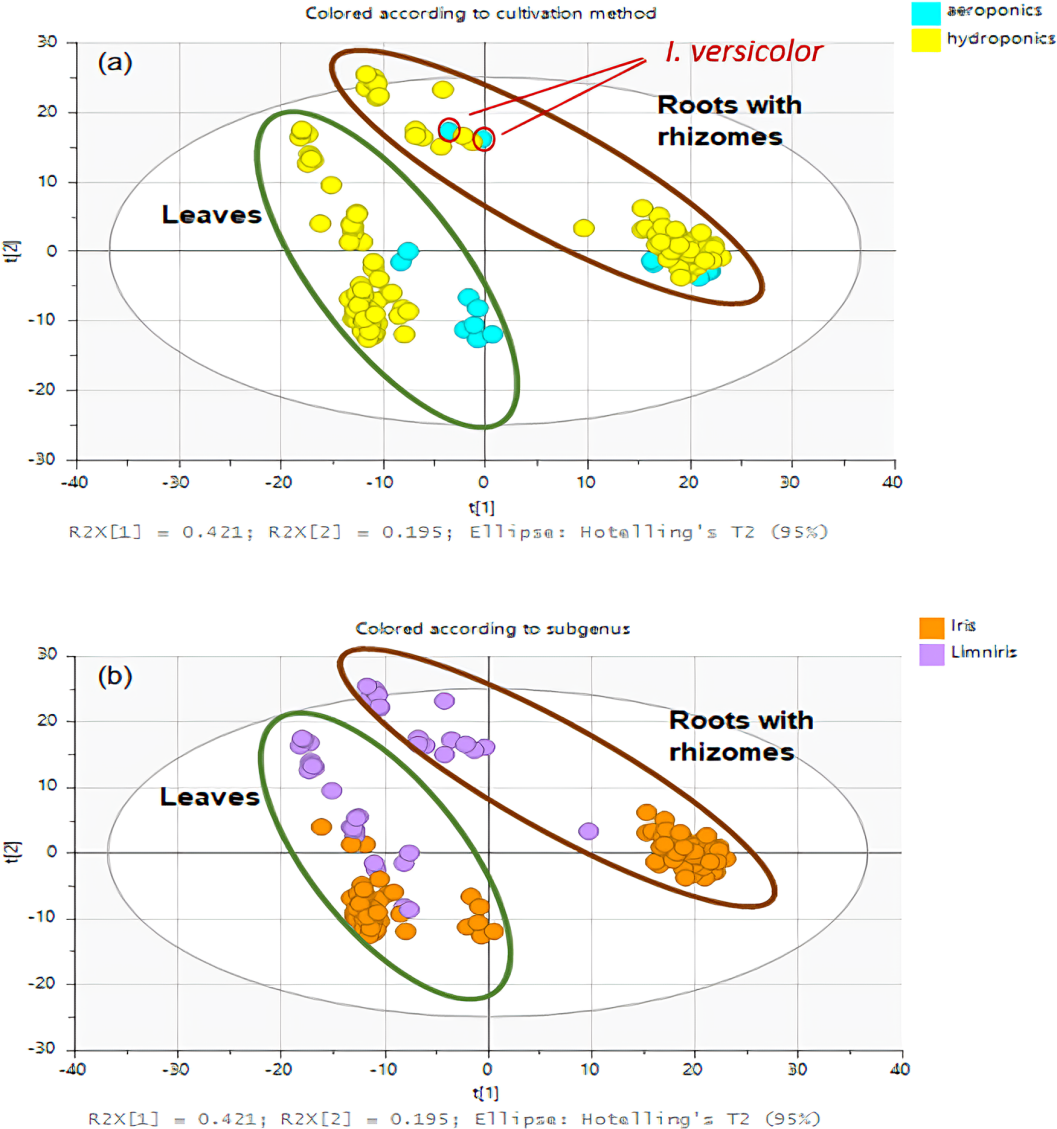
PCA graph of the data set for the analyzed *Iris* extracts. Plots colored by the (a) cultivation method and (b) subgenus, with highlighted plant part separation.

### 2.1 *Iris* Subgenera, Species, and Cultivars

The *Iris* species and cultivars analyzed in this study were representatives of the subgenera *Iris* and *Limniris*, the former characterized by the presence of a beard on the flower petals, and the latter by a crest.^45^ As demonstrated earlier, clustering of samples based on the subgenus was observed, justifying further investigation. A Volcano plot was used to reduce the normalized data matrix from all samples, filtering 504 significant metabolites, 395 downregulated and 109 upregulated in the *Iris*/*Limniris* direction (Supporting Information, S4). The supervised Orthogonal Partial Least Squares Discriminant Analysis (OPLS-DA) applied to the reduced data set achieved a clear separation, with the model achieving an excellent predictive value (*Q* ^2^ = 0.90). Table 1 details 10 compounds with the highest VIP scores, 5 for each subgenus, that were identified as possible chemotaxonomic markers. Of these, 8 compounds were (iso)­flavonoids and 2 were phenols, all detected in both leaves and roots with rhizomes. Some of the proposed compounds were previously reported to be bioactive, such as the antioxidant irilone 4′-*O*-beta-d-glucopyranoside^46^ or the anticarcinogenic and anti-inflammatory eupatorin.^47^ To explore tissue-specific chemotaxonomic markers, a Volcano plot analysis was conducted within leaves and roots with rhizomes separately (Supporting Information, S4). Compounds that were not detected in a given plant part were excluded from the analysis for that part, as including them could produce artifacts: missing values are typically imputed during preprocessing, which can artificially inflate fold changes and statistical significance.

**Table 1:** Chemotaxonomic Markers of Subgenus *Iris* and Limniris

marker	compound	VIP	tentative identification^a^	compound group^b^	chemical formula	RT (min)	plant part^c^	ionization polarity	dominant ion/searched *m*/*z*	top fragment ions	reference MS/MS^d^
Limniris	8	1.92	2,6,4′-trihydroxy-4-methoxybenzophenone	benzophenone	C_14_H_12_O_5_	3.93	L, R	NEG	[M + HCOO]^−^/305.0667	125.0258, 137.0243, 167.0338, 221.0457, 121.0672	n.a.
	109	1.75	alpinone	flavanonol	C_16_H_14_O_5_	11.68	L, R	NEG	[M – H]^−^/285.0768	124.0168, 139.0408, 117.0350, 179.0325, 97.0309	n.a.
	233	1.74	irilone/irisone B	isoflavonoid	C_16_H_10_O_6_	12.72	L, R	NEG	[M – H]^−^/297.0405	282.0157, 253.0138, 226.0273, 181.0373, 198.0309	^10^ irisone B n.a.
	108	1.62	alpinone	flavanonol	C_16_H_14_O_5_	10.87	L, R	NEG	[M – H]^−^/285.0768	119.0500, 116.0194, 93.0337, 65.0013, 97.0290	n.a.
	297	1.61	isoorientin/orientin/kaempferol 3-*O*-galactoside/kaempferol 3-*O*-glucoside	flavone glycoside	C_21_H_20_O_11_	7.56	L, R	NEG	[M – H]^−^/447.0988	n.a.	n.a.
*Iris*	607	1.41	irisjaponin B/irigenin S	isoflavonoid	C_19_H_18_O_8_	10.25	L, R	POS	[M + H]+/375.1074	345.0591, 342.0724, 360.0821, 329.0637, 327.0482	n.a.
	98	1.37	9-methoxyirispurinol/eupatorin	12a-hydroxyrotenoid/flavone	C_18_H_16_O_7_	10.16	L, R	NEG	[M – H]^−^/343.0823	328.0587, 313.0361, 298.0101, 270.0124, 285.0413	9-methoxyirispurinol n.a.,^47^
	591	1.37	irilone 4′-*O*-beta-d-glucopyranoside	isoflavonoid glycoside	C_22_H_20_O_11_	8.48	L, R	POS	[M + H]+/461.1078	299.0549, 85.0278, 314.0643, 69.0325, 97.0275	^10^
	14	1.19	3,5-dihydroxy-4-(4-hydroxybenzoyl)phenyl hexopyranoside	benzophenone glycoside	C_19_H_20_O_10_	4.49	L, R	NEG	[M – H]^−^/407.0984	245.0435, 287.0517, 151.0004, 125.0199, 193.0116	n.a.
	21	1.14	3-*O*-methylgalangin/acacetin/genkwanin/izalpinin	flavone	C_16_H_12_O_5_	11.54	L, R	NEG	[M – H]^−^/293.0612	268.088, 240.0428, 117.0338, 195.0474, 163.0374	MoNA, massbank, izalpinin n.a.

a Tentative identification based on the in-house metabolite database and further checked against online spectral data (column Reference MS/MS); isomeric compounds may occur and are separated by “/”.

b Proposed isomers can belong to different compound groups separated by “/”.

c Occurrence of a compound in the leaves (**L**) and/or roots with rhizomes (**R**).

d Reference MS/MS spectrum was not available (**n.a**.) or not searched for (−) due to missing MS2 information for the compound in the experiment.

Research carried out until now has highlighted the taxonomic significance of certain (iso)­flavonoids with restricted occurrence in *Iris*. For example, the presence of isoflavonoid aglycones, rather than glycosides, has been noted as a characteristic feature of *Limniris*.^46,48^ This observation was partly confirmed in our study with many of the significant (iso)­flavonoid markers of *Limniris*, including those listed in Table 1, being isoflavonoid aglycones. Nevertheless, some *Limniris* markers were also suspected to be glycosides, such as compound 203 (iridin). Moreover, certain isoflavonoid aglycones were more characteristic of the subgenus *Iris*, such as compound 607 (irisjaponin B/irigenin S). Notably, when examining the sum of normalized signals for compounds within individual compound groups, the Volcano plot, included in Supporting Information, S5, revealed that isoflavonoid aglycones were not significant for distinguishing the subgenera, whereas glycosides were upregulated in *Iris*. This observation was supported by OPLS-DA, which indicated a similar distribution pattern for isoflavonoid aglycones and glycosides, with each group contributing to the differentiation between the subgenera. This suggests that the mere presence or absence of these compounds is not a reliable taxonomic marker for a subgenus classification.

On the other hand, benzoquinones and cinnamaldehydes were found to be upregulated in *Limniris*, with some compounds, like compound 264 (irisoquin A), compound 272 (irisoquin F), and compound 532 (coniferaldehyde), surpassing a VIP score of 1.2. Another distinguishing feature of *Limniris* was the upregulation of phenolic acids, with compounds 369 (protocatechuic acid) and 137 (caffeic acid) serving as the strongest markers. Additionally, sesquiterpenoids, triterpenoids, steroids, and sterols were also among the significant compound groups for characterizing the *Limniris* subgenus.

The metabolic profiles of different species varied significantly in some cases, primarily due to belonging to different subgenera, as discussed earlier. For example, a strong difference was observed between *I. germanica* (*Iris*) and *I. versicolor* (*Limniris*). In the various cultivars of *I. germanica*, (iso)­flavonoids predominated, with some of the most prominent signals attributed to compound 226 (irigenin, confirmed with an analytical reference standard), compound 429 (5,3′,4′,5′-tetramethoxy-6,7-methylenedioxyisoflavone), and compound 578 (iridin). In contrast, the (iso)­flavonoid profile in *I. versicolor* was relatively poor, with an average of 98 (iso)­flavonoids detected compared to 148 in *I. germanica*. A few (iso)­flavonoids seemed to be a characteristic of *I. versicolor*, including compound 233 (irilone/irisone B), one of the *Limniris* markers, as well as compound 187 (formononetin) and compound 200 (iridin), both of which were only detected in the leaves of *I. versicolor*. Other characteristic compounds included several tentatively identified terpenoids, such as compound 727 (spirobicyclic triterpenoid), compound 107 (a-dehydroirigermanal/missourin), compound 290, and 650 (iritectol A/22,23-epoxy-21-hydroxyiridal/iritectol B). These terpenoids were generally absent in *I. germanica* or showed weak signals. Notably, compounds 290 and 650 were detected exclusively in the aeroponically grown roots with rhizomes of *I. versicolor*, implying a significant effect of the cultivation method. In addition, *I. versicolor* displayed elevated levels of cinnamaldehydes and benzoquinones compared with other species. Interestingly, some benzoquinones extracted from *Iris* were recently linked to allelopathic effects.^49^

While some metabolites appeared to be species-specific, 40 metabolites were found across all studied species and cultivars, suggesting that they could be ubiquitous within the *Iris* genus. Among these were compound 395 (tectorigenin, confirmed by an analytical reference standard), compound 593, and compound 734, possibly irilone and tectoridin, respectively, which are considered some of the most representative compounds of the *Iris* genus [40]. Of these 40 common metabolites, 6 were detected in all samples (including both plant parts, aeroponic, hydroponic, treated, and control samples), though at varying levels. These included compound 593 (irilone/irisone B), compound 632 (irispurinol/quercetin 3,4′-dimethyl ether/iristectorigenin A), compound 429 (5,3′,4′,5′-tetramethoxy-6,7-methylenedioxyisoflavone), compound 731 (stigmasterol), compound 292 (isomangiferin/nigricanside), and compound 325 (mangiferin, confirmed by an analytical reference standard). Similar to our findings, Williams et al. (1997) reported the presence of mangiferin and isomangiferin in all 37 *Iris* species and cultivars they studied,^50^ and these *C*-glycosyl xanthones have been described in over 40 *Iris* species to date.^17^ The presented study expands on this list, confirming mangiferin in previously uninvestigated species such as *Iris nyaradyana* or *Iris neglecta*.

In general, cultivars of the same species exhibited similar metabolic profiles. This was evident from the PCA described in chapter 2, Results and discussion, where some clustering was observed among different cultivars of *I. germanica*, particularly in the roots with rhizomes. For example, *I. germanica* Florentina Coerulea (sample 61, 260 targeted metabolites) and *I. germanica* Florentina Alba (sample 53, 266 targeted metabolites) showed almost identical profiles of detected compounds, as shown in Figure 3.

**Figure 3:**
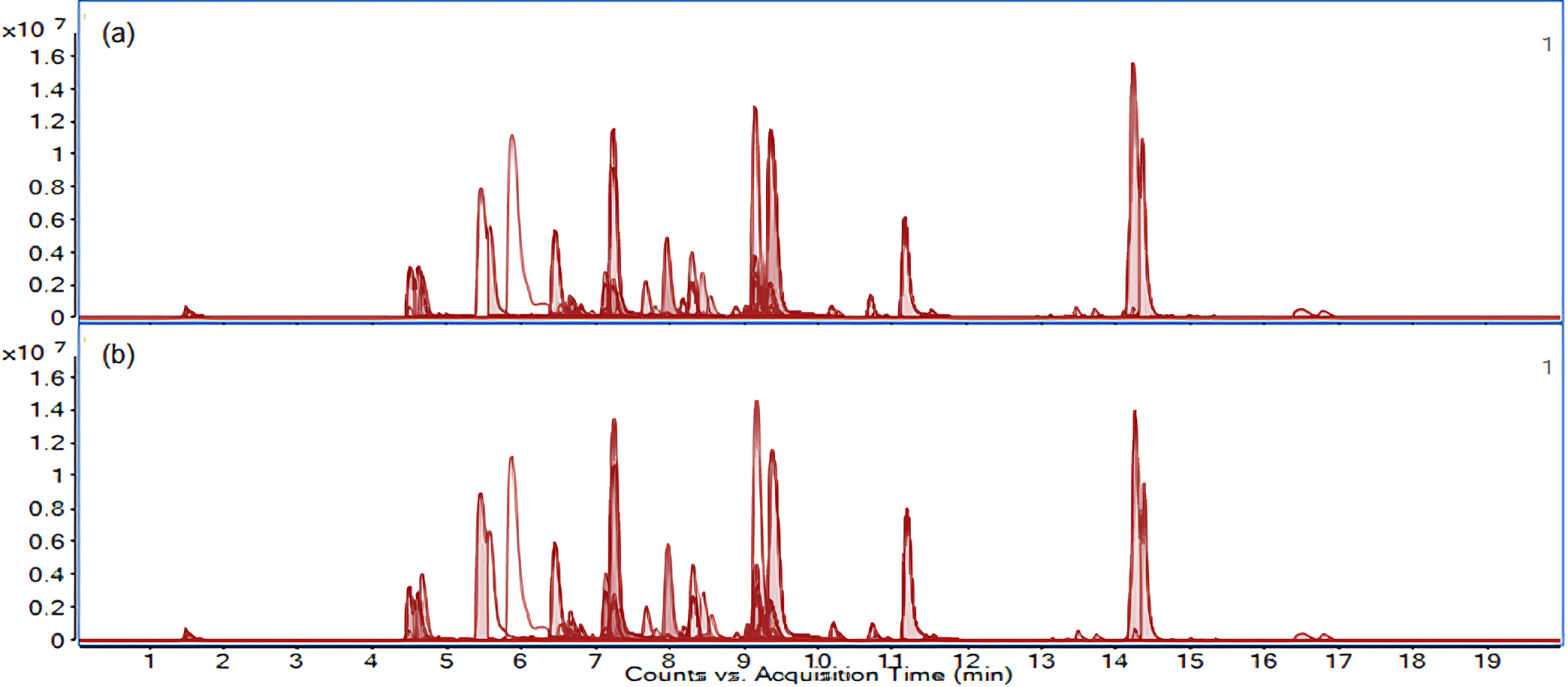
Phytochemical profile of *Iris germanica* cultivars. Extracted ion chromatograms (EIC) of compounds detected in the negative ionization mode in the extracts of roots with rhizomes of (a) *I. germanica* Florentina Coerulea and (b) *I. germanica* Florentina Alba based on the in-house metabolite database.

### 2.2 Leaves vs Roots with Rhizomes

The chemical profiles of the leaves and roots with rhizomes of the studied *Iris* species were distinctly different, as illustrated by the PCA described in chapter 2, Results and discussion. Considering the entire sample set, more targeted metabolites were identified in the roots with rhizomes (503) compared with the leaves (438), this trend being true for most species and cultivars, with 194 metabolites common to both plant parts.

According to the Volcano plot analysis included in the Supporting Information, S6, 228 metabolites showed an upregulated expression in the leaves, while 278 metabolites were upregulated in the roots with rhizomes. The upregulated compound groups in the leaves included phenolic acids, steroids, *C*-glycosyl xanthones, and flavone/flavone glycosides. In contrast, compounds, such as stilbenes, triterpenoids, flavanonol glycosides, isoflavonoids, and their glycosides, cinnamaldehydes, and benzophenones were more abundant in the roots with rhizomes. This finding aligns with the literature, which reports that isoflavonoids are primarily found in the rhizomes, while the leaves are rich in apigenin- and luteolin-based *C*- and *O*-glycosylflavones, along with their derivatives, such as vitexin, isovitexin, orientin, isoorientin, and swertisin.^46^

Subsequently, OPLS-DA was performed on the Volcano-reduced data set, generating a robust model capable of predicting the plant part with a *Q* ^2^ value of 0.99. Table 2 presents the 10 metabolites with the highest VIP score: 5 for each plant part.

**Table 2:** Differential Metabolites of the Leaves and Roots with Rhizomes of *Iris*

marker	compound	VIP	tentative identification^a^	compound group^b^	chemical formula	RT (min)	plant part^c^	ionization polarity	dominant ion/searched *m*/*z*	top fragments	reference MS/MS^d^
roots with rhizomes	182	1.79	formononetin	isoflavonoid	C_16_H_12_O_4_	8.32	R	NEG	[H + HCOO]-/313.0718	298.0472, 283.0238, 297.0370, 269.0431, 239.0398	n.a.
	220	1.76	iriflophenone	benzophenone	C_13_H_10_O_5_	5.87	L, R	NEG	[M – H]^−^/245.0455	93.0336, 107.0144, 151.0028, 65.0017, 63.0219	MoNA
	13	1.74	3′,4′,6-trimethoxy-5′,7,8-trihydroxyisoflavone	isoflavonoid	C_18_H_16_O_8_	9.21	R	NEG	[M – H]^−^/359.0772	329.0300, 344.0527, 314.0069, 286.0110, 297.0124	^51^
	599	1.72	irisdichotin A	flavonol glycoside	C_23_H_24_O_12_	7.18	L, R	POS	[M + H]+/493.1341	331.081, 316.0562	n.a.
	69	1.70	6,4′-dimethoxy-5-hydroxyflavone 7-glucoside/4′-methyltectorigenin-7-glucoside (1. conformer)/ 4′-methyltectorigenin-7-glucoside (2. conformer)/ irisolidone-7-*O*-a-d-glucoside	flavone glycoside/isoflavonoid glycoside	C_23_H_24_O_11_	7.22	L, R	NEG	[H + HCOO]-/521.1301	359.0773, 343.0467, 344.0540, 506.1073, 328.0203	n.a.
leaves	44	1.66	5,3′,4′,5′-tetramethoxy-6,7-methylenedioxyisoflavone	isoflavonoid	C_20_H_18_O_8_	6.66	L	NEG	[H + HCOO]-/431.0984	311.0503, 341.0624, 283.0582, 323.0508, 281.0396	n.a.
	391	1.62	swertisin/4′-*O*-methylapigenin 6-*C*-hexoside/4′-*O*-methylapigenin 8-*C*-hexoside	flavone glycoside	C_22_H_22_O_10_	6.83	L	NEG	[M – H]^−^/445.1140	297.0383, 325.0696, 282.0514, 231.0297, 117.0305	MoNA^12^
	438	1.61	5,7-dihydroxy-6-methoxychromon	chromone	C_10_H_8_O_5_	1.79	L	POS	[M + H]+/209.0444	n.a.	
	82	1.61	7-*O*-methylmangiferin/irisxanthone/7-*O*-methylisomangiferin	*C*-glycosyl xanthone	C_20_H_20_O_11_	6.44	L	NEG	[M – H]^−^/435.0933	315.0516, 345.0615, 272.0330, 330.0377, 300.0260	^51^, irishanthone n.a.
	295	1.61	isomangiferin/nigricanside	*C*-glycosyl xanthone	C_19_H_18_O_11_	7.16	L	NEG	[M – H]^−^/421.0776	259.0205, 301.0323, 331.0435, 227.0271, 313.0333	^51^

a Tentative identification based on the in-house metabolite database and further checked against online spectral data (column Reference MS/MS); isomeric compounds may occur and are separated by “/”.

b Proposed isomers can belong to different compound groups separated by “/”.

c Occurrence of a compound in the leaves (**L**) and/or roots with rhizomes (**R**).

d Reference MS/MS spectrum was not available (**n.a**.) or not searched for (−) due to missing MS2 information for the compound in the experiment.

### 2.3 Aeroponics vs Hydroponics

As mentioned in chapter 1, Introduction, the cultivation method influences the availability and uptake of nutrients by plants and can also induce stress, which in turn may alter the profile of secondary metabolites. In this study, we evaluated the differences in the profiles of detected metabolites between all samples of aeroponically and hydroponically grown irises.

The Volcano plot analysis conducted on the whole data set revealed that 232 metabolites were upregulated in hydroponically grown plants, while only 42 metabolites showed upregulation in aeroponically grown plants (Supporting Information, S7). This leads to the presumption that hydroponics, under the conditions used in the experiment, is more effective for the enhanced production of a broader range of *Iris* metabolites. Chromones, anthocyanins, flavone, and isoflavonoid glycosides were among the compound groups most upregulated in hydroponics, whereas in aeroponics, groups such as xanthones and benzofurans showed a stronger response.

Subsequently, supervised OPLS-DA was applied to identify the secondary metabolites most significantly influenced by the cultivation method. The model successfully discriminated between the two groups with a predictive ability *Q* ^2^ = 0.90. Table 3 details 10 compounds with the highest VIP score, 5 for aeroponics, and 5 for hydroponics.

**Table 3:** Differential Metabolites of *Iris* Cultivated in Aeroponics and Hydroponics

marker	compound	VIP	tentative identification^a^	compound group^b^	chemical formula	RT (min)	plant part^c^	ionization polarity	dominant ion/searched *m*/*z*	top fragments	reference MS/MS^d^
hydroponics	439	1.85	5,7-dihydroxy-6-methoxychromon	chromone	C_10_H_8_O_5_	2.03	L, R	POS	[M + NH4]+/226.0710	97.0264, 84.0440, 180.0626, 106.0635, 121.0729	n.a.
	78	1.84	7-*O*-methylaromadendrin/hesperetin/dihydrokaempferide/5,7,4′-trihydroxy-6-methoxyflavanone	flavanone/flavanonol	C_16_H_14_O_6_	9.83	L, R	NEG	[M-H]-/301.0718	n.a.	
	276	1.75	irispurinol/quercetin 3,4′-dimethyl ether/iristectorigenin A	12a-hydroxyrotenoid/flavone/isoflavonoid	C_17_H_14_O_7_	9.60	L, R	NEG	[M-H]-/329.0667	n.a.	
	316	1.57	isovitexin/vitexin	flavone glycoside	C_21_H_20_O_10_	7.41	L	NEG	[H + HCOO]-/477.1038	n.a.	n.a.
	529	1.55	cinnamic acid	phenolic acid	C_9_H_8_O_2_	5.64	L, R	POS	[M + NH4]+/166.0863	80.0482, 136.0758, 78.0323, 53.0368, 69.0322	n.a.
aeroponics	589	1.97	irilone 4′-*O*-beta-d-glucopyranoside	isoflavonoid glycoside	C_22_H_20_O_11_	7.72	L, R	POS	[M + H]+/461.1078	299.0551, 85.0276, 69.0329, 341.0628, 127.0372	^10^
	524	1.87	betavulgarin/irisolone/irisone A	isoflavonoid	C_17_H_12_O_6_	9.61	L, R	POS	[M + H]+/313.0707	298.0475, 297.0398, 180.0053, 240.0404, 270.0515	n.a.
	114	1.82	alpinone	flavanonol	C_16_H_14_O_5_	8.71	L, R	NEG	[H + HCOO]-/331.0823	165.9913, 316.0584, 110.0016, 273.0392, 82.0056	n.a.
	684	1.79	kanzakiflavone-1/iriflogenin/soforanarin A/irisoid A	flavone/isoflavonoid/peltogynoid	C_17_H_12_O_7_	8.83	L, R	POS	[M + H]+/329.0656	314.0408, 180.0054, 297.0395, 269.0440, 301.0681	n.a.
	321	1.77	luteolin-4′,7-dimethyl ether/irilin A/irisolidone/5,7-dihydroxy-2′,6-dimethoxyisoflavone	flavone/isoflavonoid	C_17_H_14_O_6_	7.24	L	NEG	[H + HCOO]-/359.0772	329.0295, 344.0535, 314.0056, 230.0187, 285.0407	n.a.

a Tentative identification based on the in-house metabolite database and further checked against online spectral data (column Reference MS/MS); isomeric compounds may occur and are separated by “/”.

b Proposed isomers can belong to different compound groups separated by “/”.

c Occurrence of a compound in the leaves (**L**) and/or roots with rhizomes (**R**).

d Reference MS/MS spectrum was not available (**n.a**.) or not searched for (−) due to missing MS^2^ information for the compound in the experiment.

Although the hydroponic experiment encompassed a substantially larger number of samples and species than the aeroponic experiment, analyses were performed on the combined data set to maximize statistical power and robustness. Both cultivation modes included representatives of the *Iris* and *Limniris* subgenera, which partially controlled for potential phylogenetic bias. To validate the results, a paired Volcano plot analysis (Supporting Information, S7) was conducted on the subset of common samples, showing that 90% of the significant metabolites were also significant in the full data set, reinforcing the reliability of the applied approach. The use of the full data set also enabled the tissue-specific evaluation of the cultivation mode, which, while technically feasible with the limited overlap of species and cultivars, would have lacked statistical strength and representativeness. In both leaves and roots with rhizomes, the trend of hydroponics to enhance the production of a larger number of targeted *Iris* metabolites was confirmed. Up- and downregulated metabolites, as determined by Volcano plots, are presented in Supporting Information, S7. As explained earlier, compounds not detected in a given plant part were excluded from the analysis for that part to avoid reporting metabolites with an apparent statistical significance arising from the missing value imputation.

### 2.4 Supportive Plant Treatment during Growth

The effect of treatment with a chicken feather hydrolysate on the profile of targeted metabolites was examined in aeroponically grown plants. According to the Volcano plot analysis, no significant differences were observed between the profiles of detected metabolites of the treated plants and the control group. While the feather hydrolysate provided additional amino acids and peptides, the plants were simultaneously fertilized. It is possible that the fertilization supplied all of the essential nutrients needed for optimal plant growth, making the feather hydrolysate redundant in this experimental setup and offering no additional benefit over the standard fertilizer. Future studies should explore the use of a feather hydrolysate as the sole nutrient source to determine its effectiveness as a cost-effective growth medium while also addressing feather waste disposal and potentially supporting a circular economy.

The effect of inoculation with arbuscular mycorrhizal fungi on the profile of targeted metabolites was investigated in hydroponic samples. Comparing the treated samples and controls, the Volcano plot revealed no significantly up- or downregulated metabolites. This finding is in contrast with the general trend reported in the literature, where plant treatment with AMF is commonly associated with the increased production of biomass and secondary metabolites, such as terpenoids, phenolics, alkaloids, and various flavor compounds.^52–54^ Only a few studies reported no significant effect or even a decrease in the biosynthesis of certain secondary metabolites.^55,56^ For example, Crişan et al. (2019) observed that AMF inoculation enhanced the production of some metabolites in certain *I. germanica* cultivars while having no significant effect on others.^57^

In our study, the absence of a detectable metabolite response may be due to several factors. First, colonization appeared relatively weak in many plants, limiting the potential impact of AMF. Second, compatibility between the plant genotypes and the specific AMF strain used may have been suboptimal, preventing the establishment of a symbiotic relationship. Third, the hydroponic environment may reduce the reliance of plants on AMF-mediated nutrient intake, thereby attenuating the metabolic response; consequently, AMF treatment could exert different effects in conventional cultivation. Additional environmental factors, such as media composition, nutrient balance, and the presence of growth regulators, may also have influenced the outcome.^58,59^ Importantly, however, AMF-treated plants still exhibited increased antimicrobial and cytotoxic activity, indicating that the symbiosis may have modulated metabolic pathways, leading to metabolic products beyond the scope of our targeted metabolites. In spite of our extensive in-house database of *Iris* metabolites, other constituents, including mycorrhizal-derived metabolites, may underlie the observed bioactivity, providing a rationale for follow-up studies, such as nontargeted metabolomic screening to identify these differential compounds.

### 2.5 Identification of Potential Bioactive Metabolites

In addition to the phytochemical analysis, the *Iris* extracts were tested for cytotoxic, antimicrobial, anti-inflammatory, and antioxidant activities, evaluated using human dermal fibroblasts and melanoma cells, selected microorganisms (*Staphylococcus aureus*, *Salmonella enterica*, *Cutibacterium acnes*, *Candida albicans*), RAW 264.7 macrophages, and the ORAC assay, respectively, following established protocols as detailed in Section 4.3, Bioactivity assays. Many of the extracts showed strong bioactive properties. To identify potential bioactive compounds, the bioassay data and normalized metabolite signals obtained through the targeted UHPLC-HRMS/MS screening of secondary metabolites were correlated using the Pearson correlation, as described in Section 4.7 Chemometric analysis. The data matrix used for the correlational analysis is presented in Supporting Information, S8. All samples, including those exhibiting no detectable bioactivity, were incorporated into the correlation analysis to ensure an unbiased evaluation of the relationships between the chemical composition and biological activity. A moderate correlation was found between some individual metabolites and cytotoxic, anti-inflammatory, and antimicrobial activity (inhibition of *C. albicans* and *S. enterica*). The details of these metabolites are listed in Table 4. Figure 4 depicts the structures of selected metabolites that to our knowledge are associated for the first time with the observed bioactivity.

**Table 4:** Compounds Correlated to the Tested Bioactivities

bioactivity	compound	*r*	tentative identification^a^	compound group^b^	chemical formula	RT (min)	plant part^c^	ionization polarity	dominant ion/searched *m*/*z*	top fragments	reference MS/MS^d^
cytotoxic	37	0.44	4′,7-di-*O*-methyldihydroquercetin/5,7,3′-trihydroxy-6,4′-dimethoxyflavanone	flavone	C_17_H_16_O_7_	9.03	R	NEG	[M – H]^−^/331.0823	n.a.	
	89	0.41	8-hydroxyirigenin	isoflavonoid	C_18_H_16_O_9_	8.18	R	NEG	[M – H]^−^/375.0722	360.0493, 345.0252, 301.0346, 330.0001, 204.9776	n.a.
	206	0.44	iriflophenone 2-*O*-rhamnoside	benzophenone	C_19_H_20_O_9_	4.70	R	NEG	[M + HCOO]-/437.1089	317.0630, 275.0511, 301.0934, 335.7683, 367.8801	n.a.
	358	0.42	phalerin	benzophenone	C_20_H_22_O_10_	6.23	R	NEG	[M-H]-/421.1140	301.0705, 301.0340, 331.0809, 215.0698,258.0160	n.a.
	387	0.43	swertiajaponin/tectoridin/tectorigenin-4′-*O*-beta-glucoside	flavone glycoside/isoflavonoid/isoflavonoid glycoside	C_22_H_22_O_11_	6.77	R	NEG	[M – H]^−^/461.1089	n.a.	
	405	0.43	1,11-dihydroxy-9,10-methylenedioxy-12a-dehydrorotenoid/irisoid D	12a-dehydrorotenoid/peltogynoid	C_17_H_10_O_7_	4.06	R	POS	[M + NH_4_]^+^/344.0765	185.0503	n.a.
	459	0.43	7-beta-hydroxystigmast-4-en-3-one	steroid	C_29_H_48_O_2_	13.76	R	POS	[M + H]^+^/429.3727	n.a.	
	466	0.41	hesperetin/dihydrokaempferide/5,7,4′-trihydroxy-6-methoxyflavanone	flavanone/flavanonol/flavanone	C_16_H_14_O_6_	8.98	R	POS	[M + H]^+^/303.0863	153.0175, 145.0281, 177.0542, 117.0344, 149.0574	HMDB, dihydrokaempferide n.a., 5,7,4′-trihydroxy-6-methoxyflavanone n.a.
	471	0.48	irisxanthone	*C*-glycosyl xanthone	C_20_H_20_O_11_	6.53	R	POS	[M + H]^+^/437.1078	287.0550, 317.0651, 341.0648, 353.0644, 313.0695	MoNA
	488	0.50	a-dehydroirigermanal/missourin	triterpenoid	C_30_H_48_O_3_	13.76	R	POS	[M + H]+/457.3676	109.1007, 177.1633, 159.1170, 439.3555, 95.0846	n.a.
	518	0.44	beta-sitosterol/24-methylpollinasterol	sterol	C_29_H_50_O	14.42	R	POS	[M + H]^+^/415.3934	n.a.	
	578	0.40	iridin	isoflavonoid glycoside	C_24_H_26_O_13_	7.29	L, R	POS	[M + H]^+^/523.1445	361.0918, 346.0677	^60^
	601	0.44	irisjaponin A	isoflavonoid	C_20_H_20_O_9_	10.10	R	POS	[M + H]^+^/405.1180	n.a.	
	614	0.44	iriskumaonin methyl ether	isoflavonoid	C_19_H_16_O_7_	10.29	L, R	POS	[M + H]^+^/357.0969	342.0729, 327.0489, 341.0650, 299.0537, 220.0360	n.a.
	722	0.44	*p*-hydroxybenzoic acid/salicylic acid	phenolic acid	C_7_H_6_O_3_	4.52	R	POS	[M + H]^+^/139.0390	n.a.	
antibacterial (C. albicans)	337	0.42	*p*-coumaric acid	phenolic acid	C_9_H_8_O_3_	5.61	R	NEG	[M + HCOO]-/209.0455	119.0499, 91.0184, 104.0182, 93.0339, 117.0322	n.a.
antibacterial (S. enterica)	174	0.41	*E*-ferulic acid/Z-ferulic acid	phenolic acid	C_10_H_10_O_4_	7.58	L	NEG	[M – H]^−^/193.0506	133.0288, 134.0373, 80.3851	MoNA, *Z*-ferulic acid n.a.
anti-inflammatory	146	0.41	*cis*-vaccenic acid	fatty acid	C_18_H_34_O_2_	14.60	L	NEG	[M – H]^−^/281.2486	n.a.	
	392	0.52	swertisin/4′-*O*-methylapigenin 6-*C*-hexoside/4′-*O*-methylapigenin 8-*C*-hexoside	flavone/flavone glycoside/flavone glycoside	C_22_H_22_O_10_	7.12	L	NEG	[M + HCOO]^−^/491.1195	n.a.	
	740	0.40	tlatancuayin	isoflavonoid	C_18_H_14_O_6_	11.09	L	POS	[M + H]+/327.0863	234.1028, 312.0653, 257.1908, 151.0529, 312.1724	n.a.

a Tentative identification based on the in-house metabolite database and further checked against online spectral data (column Reference MS/MS); isomeric compounds may occur and are separated by “/”.

b Proposed isomers can belong to different compound groups separated by “/”.

c Occurrence of a compound in the leaves (**L**) and/or roots with rhizomes (**R**).

d Reference MS/MS spectrum was not available (**n.a**.) or not searched for (−) due to missing MS^2^ information for the compound in the experiment.

**Figure 4:**
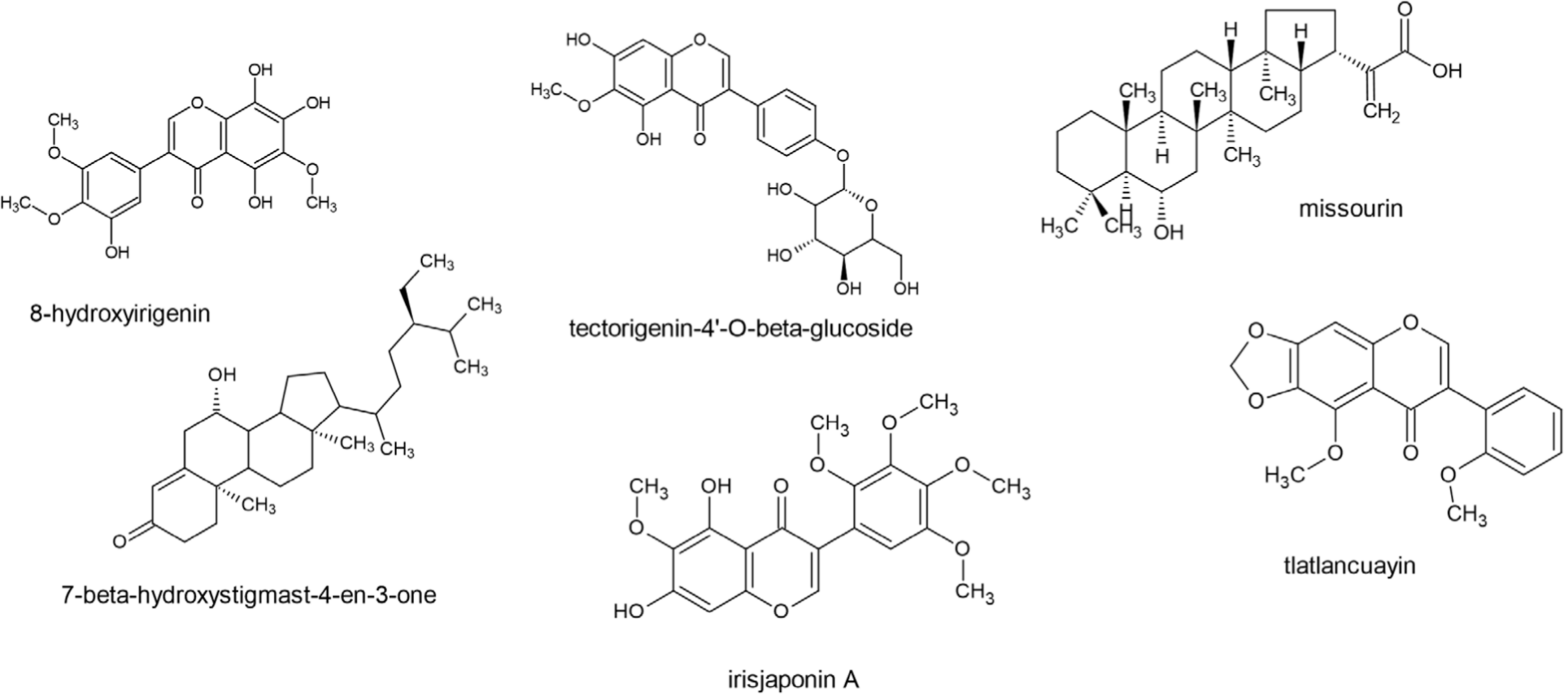
Tentative chemical structures of selected *Iris* metabolites correlated to bioactivity.

Altogether, 15 metabolites showed a moderate correlation with the cytotoxic activity. Among them was compound 206, tentatively identified as iriflophenone 2-*O*-rhamnoside, previously reported to exhibit significant toxicity against human tumor cells,^61^ compound 358, tentatively identified as phalerin, which has shown inhibitory effects against myeloma and leukemia cell lines,^62^ and compound 578, tentatively identified as iridin, described as possessing promising anticarcinogenic properties.^63^ Some of the correlated compounds lack bioactivity data and are thus candidates for further testing. Most of the compounds were detected in the roots with rhizomes only or showed a strong upregulation in this plant part, such as iridin (compound 578), indicating that the roots with rhizomes contain metabolites that the warrant closer examination for potential cytotoxic activity. Nonetheless, it should be stressed that these correlations do not establish causality, and additional experimental validation through compound isolation and testing is essential, following approaches such as Turk et al. (2025) in their study on the antidiabetic potential of purified compounds from *Iris palaestina* following nontargeted metabolomic profiling.^73^

Two phenolic acids were correlated to antimicrobial activity. Compound 337, believed to be *p*-coumaric acid, was associated with the inhibition of *C. albicans*, confirming its previously reported antifungal properties.^64,65^ This compound was detected only in the roots with rhizomes. Ferulic acid (compound 174), which was a characteristic of the leaves of *Iris*, has been linked to the inhibition of *S. enterica*, consistent with findings from earlier research.^66^

Concerning the anti-inflammatory activity, three detected metabolites showed a moderate correlation, including compound 146—*cis*-vaccenic acid, which has previously been reported to modulate inflammatory processes in human cells.^67–69^ These compounds were detected only in the leaves, indicating that the leaves of *Iris* may contain compounds with potential anti-inflammatory relevance.

No antibacterial effect was observed against *S. aureus* in the bioactivity assay. While several extracts demonstrated antioxidant properties and antibacterial effects against *Propionibacterium acnes*, no single metabolite detected in this study was found to be significantly correlated. It should be emphasized that the observed bioactive properties of the *Iris* extracts may be the result of synergistic interactions of the complex mixture of metabolites rather than the action of an individual compound. The synergistic or antagonistic activity of constituents of plant extracts has been well described, owing to multitarget mechanisms of action as well as effects on solubility, resorption, and bioavailability.^70,71^ Another key consideration is that the Pearson correlation is designed for linear relationships, so it may not effectively capture compounds exhibiting nonlinear bioactive effects.^72^

Overall, the presented results should be viewed as preliminary and serve as a platform for further research. The possibility of false positives inherent in correlation analyses of complex crude extracts should also be acknowledged, and the isolation and purification of candidate bioactive metabolites are essential to confirm causality through detailed bioactivity testing.

## 3 Conclusion

*Iris* plants occupy an important place in agriculture, horticulture, traditional medicine, and pharmacology. In this study, we characterized the phytochemical profiles of a unique collection of various *Iris* species and cultivars grown under aeroponic and hydroponic conditions. The leaves and roots with rhizomes displayed distinct patterns of secondary metabolites, including phenolic acids, stilbenes, terpenoids, and other compounds, many of which were significantly up- or downregulated. The cultivation system itself markedly influenced metabolite accumulation, with hydroponic growth leading to a significant upregulation of numerous compounds. In contrast, treatments with arbuscular mycorrhizal fungi or feather hydrolysate did not produce notable changes in the targeted metabolite profiles. Nonetheless, AMF-treated plants exhibited increased antimicrobial and cytotoxic activities, suggesting that other unprofiled compounds may contribute to this effect. Several identified metabolites may serve as chemotaxonomic markers, and some show potential cytotoxic, anti-inflammatory, and antimicrobial properties. Overall, these findings highlight the potential of soil-less cultivation systems to enhance the production of valuable phytochemicals in *Iris* species. Moreover, the identification of distinctive chemotaxonomic markers and potential bioactive compounds provides a foundation for future research and applications in pharmacology, plant breeding, and beyond.

## 4 Materials and Methods

### 4.1 Plant Material, Cultivation, and Preparation of Crude Extracts

Leaves and roots with rhizomes of various *Iris* species and cultivars from subgenus *Iris* and *Limniris* were obtained from aeroponically and/or hydroponically grown plants in the facilities of the Institute of Botany of the Czech Academy of Sciences in Pruhonice, Czech Republic.^74^ Plants were harvested in 2023 following three years of cultivation, with each cultivated in triplicate. In total, 37 different *Iris* plants were represented: *Iris pallida* “Dalmatica”, *Iris x germanica* “ALBA”, *Iris x germanica* “Florentina Alba”, *Iris x germanica* “NEPALENSIS”, *Iris x germanica* “TBILISI”, *Iris x germanica* “VIKOS”, *Iris x germanica* “KHARPUT”, *Iris x germanica* “CRETAN”, *Iris x germanica* “BEX”, *Iris x germanica* “KOCHII”, *Iris x germanica* “Florentina Coerulea”, *Iris pallida* “KOTOR”, *Iris pallida* “MOSTAR”, *Iris croatica*, *Iris x nyaradyana*, *Iris variegata*, *Iris mediterranea*, *Iris pallida* subsp. *illyrica*, *Iris x neglecta*, *Iris mesopotamica*, *Iris x squalens*, *Iris x sambucina*, *Iris trojana*, *Iris imbricata*, *Iris albertii* “HAKAN”, *Iris x barbata* “ZUA”, *Iris x barbata* “ADMIRAL”, *I. versicolor*, *Iris pseudacorus* “GUBIJN”, *Iris sanguinea*, *Iris tectorum*, *Iris spuria* subsp. *carthaliniae*, *Iris sibirica* “CAMBERLEY”, *Iris spuria* subsp. *musulmanica*, *Iris spuria* “ROZEVLÁTÝ”, *Iris pseudacorus*, *Iris* *lactea*.

For the aeroponics experiment, plants were cultivated in a growth chamber equipped with an ultrasonic steam generator and a misting device. Fertilization was carried out using a 2% solution of Kristalon Fruit and Flower (mineral ratio nitrogen/phosphorus/potassium 15:5:30 + 3% MgO + 5% SO_3_), applied via the misting system. To maintain hygiene and prevent contamination, hydrogen peroxide (30%) was used as a disinfectant at a concentration of 5 mL/10 L of nutrient solution. *Iris* plants were grown under a 300 W LED lamp, providing an intensity of approximately 5000–6000 lx at a distance of 70 cm, with a photoperiod of 18 h of light and 6 h of darkness. Each cultivar included in the aeroponic experiment was planted five times with subsequent treatment with a feather hydrolysate and five times without. The feather hydrolysate with an initial amino acid and peptide content of 2.8% was applied as a 1% foliar spray with a wetting agent, until runoff. A total of four applications were carried out.

For the hydroponics experiment, plants were cultivated in the botanical garden in plastic pots filled with washed pumice (1 and 6 mm fraction), with a 3 cm layer of pebbles at the bottom for drainage. For each cultivar, ten rhizomes were planted, five with mycorrhizal inoculation (*Rhizophagus intraradices* BEG 140, 1000 spores per pot) and five without. Pots were placed in four beds covered with a black weed-suppressing fabric. In the first year, all plants were fertilized every 6 weeks from March with slow-release Osmocote Topdress, applied to the pumice surface. *Iris* subgenus *Iris* was also treated with dolomitic limestone in February. In the second year, a single fertilization in May was sufficient. Irrigation for *Limniris* was applied to the pumice surface every 2 days, with excess water collected in trays for the capillary uptake. *Iris* subgenus *Iris* was watered every 4–5 days, depending on the temperature. Water was applied directly to the pumice, not the foliage. To prevent rhizome overheating, pots were wrapped in a white nonwoven fabric. *Limniris* plants were additionally shaded laterally with 50 cm high white nonwoven screens. Standard horticultural care included the removal of dry or diseased leaves, rotting rhizomes, and manual weeding. An overview of the experimental *Iris* samples is provided in Supporting Information, S1.

Following the collection, the corporate partner EcoFuel Laboratories s.r.o. processed the samples into extracts. Each sample (50 g) was first disintegrated into small pieces, grated, dried at 40 °C for 48 h, and then ground into a fine powder. The powder was macerated in 80% methanol in water (*v*/*v*) for 24 h at room temperature, using a 1:15 sample-to-solvent weight ratio. The resulting crude extract was centrifuged and filtered through an organza.

### 4.2 Chemicals and Reagents

For the chemical analysis, LC-grade methanol and ammonium formate (purity ≥ 99.9%) and formic acid (purity ≥ 98%) were sourced from Merck (Germany). Distilled water was obtained by using a Milli-Q RG system from Millipore (Germany). The analytical reference standard of tectorigenin (purity of 99.58%) was purchased from Santa Cruz Biotechnology (United States). Standards of mangiferin (purity of 98.4%), irigenin (purity of 100%), and chrysoeriol (purity of 99.1%) were supplied by Cayman Chemical (United States).

For the bioactivity assays, the following standards or chemicals were purchased from Sigma-Aldrich (United States): 100× antibiotic antimycotic solution, Dulbecco’s Modified Eagle’s medium (DMEM), high glucose, Eagle’s minimum essential medium (EMEM), dimethyl sulfoxide (DMSO), l-glutamine solution, fetal bovine serum (FBS), trypsin-2,2′,2″,2‴-(ethane-1,2-diyldinitrilo)­tetraacetic acid (EDTA) solution, and resazurin sodium salt.

### 4.3 Bioactivity Assays

The crude extracts were evaporated and dissolved in DMSO to a concentration of 100 mg/mL. These extracts were used to determine the biological activity, namely, cytotoxic, antimicrobial, anti-inflammatory, and antioxidant. The maximum concentration tested was always 1 mg/mL, corresponding to the addition of a maximum of 1% DMSO. This solvent was chosen because of its low evaporation in the Biohazard box with laminar air flow. High-throughput assays were used for testing: a 96-channel Biomek FXp pipetting head station (Beckman Coulter, USA), a MultiFlow dispenser (BioTech, USA), a wash station HydroSpeed (Tecan, Switzerland), and a SpectraMax MiniMax i3x multimodal reader (Molecular Devices, USA).

Cytotoxic activity was tested as the ratio of the concentration inhibiting half of the population of the control cell line (human dermal fibroblasts, HDF, Sigma-Aldrich) to the concentration inhibiting half of the population of melanoma cells (B16, CCL-6322TM, and ATCC). The assay was performed according to Szemerédi et al. (2021),^75^ including 72 h incubation and assessment of cell viability by the resazurin assay.

Antioxidant activity was determined as the radical scavenging capacity of the extracts in the classical oxygen radical absorption capacity assay (ORAC) according to Viktorová et al. (2019).^76^

Antimicrobial activity was tested using Gram-positive and Gram-negative bacteria (*S. aureus* CCM 4223 and *S. enterica* CCM 3807, respectively), anaerobic bacteria (*Cutibacterium acnes* CCM 7417), and yeast (*C. albicans* DBM2186) according to the ISO 20776-1 standard.

Anti-inflammatory activity was determined as the ability to inhibit the production of the cytokine TNFα in the macrophages RAW 264.7 (Sigma-Aldrich) according to Tran et al. (2023).^77^

Cytotoxic activity was expressed as the therapeutic index (TI = IC_50_ HDF/IC_50_ B16), with higher TI indicating stronger selectivity. Antioxidant potential was measured as the IC_50_ value in the ORAC assay. Antimicrobial activity was determined as the reduction of microbial viability at 1 mg/mL extract; when the cell viability fell below 50%, the IC_50_ value was calculated. Anti-inflammatory activity was assessed as the inhibition of TNFα production in bacterial lipopolysaccharide-stimulated RAW 264.7 macrophages (1 mg/mL extract). To enable correlational analysis (see Section 4.7 chemometric analysis), an arbitrary scale was applied to standardize assay results. For the antimicrobial assay, scores were assigned as follows: ≥ 80% (no effect) = 1, 70–80% = 2, 60–70% = 3, 50–60% = 4, and <50% or IC_50_ values (strongest effect) = 5. For the anti-inflammatory assay, values ≥ 80% could not be directly analyzed and were similarly categorized: ≥80% = 1, 70–80% = 2, 60–70% = 3, and 50–60% (strongest effect) = 4.

### 4.4 UHPLC-HRMS/MS Analysis

The crude extracts were filtered using a 0.22 μm microfilter in a centrifuge at 5000 rpm for 2 min, and the resulting filtrate was transferred to a 2 mL vial for UHPLC-HRMS/MS analysis. The 10x diluted samples, prepared using 80% MeOH in water (*v*/*v*), were also analyzed and used in data processing for compounds with saturated peaks. Quality control (QC) samples were prepared separately for each plant part and cultivation method by taking an aliquot of the respective samples.

The samples were analyzed in a randomized order with repeated injections of blank samples (80% MeOH) and QC samples to moderate the instrument response and allow for retention time alignment. The analysis was conducted as described in our previous study^44^ on the Agilent Technologies 1290 Infinity II LC system using the ACQUITY BEH C18 (150 × 2.1 mm; 1.7 μm) column from Waters, operated at 40 °C. The injection volume was set to 3.0 μL. Mobile phase A consisted of water + formic acid (0.1%) + ammonium formate (5 mM) and mobile phase B consisted of methanol + formic acid (0.1%) + ammonium formate (5 mM). The mobile phase gradient was: 0–1 min, 95% A, 0.25 mL/min; 1–2 min, 95–70% A, 0.25 mL/min; 2–13 min, 70–0% A, 0.25 mL/min; 13–18 min, 0% A, 0.35 mL/min, with a final column equilibration time of 2 min using the initial conditions. Agilent 6560 Ion Mobility quadrupole-time-of-flight (Q-TOF) MS, operated in both positive (POS) and negative (NEG) ionization modes was used as the mass analyzer. The MS conditions were as follows: mass range 100–1200 *m*/*z*; drying gas flow 12 L/min; drying gas temperature 300 °C; sheath gas flow 12 L/min; sheath gas temperature 370 °C; nozzle voltage ± 400 V; capillary voltage ± 3500 V; and nebulizer 25 psig. MS/MS spectra were obtained by the iterative AutoMS/MS acquisition mode using 3 repeated injections of the QC samples as follows: mass range 50–1100 *m*/*z*; acquisition rate 3 spectra/s (MS) and 12 spectra/s (MS/MS); collision energy 20 eV; 5 precursor ions/cycle.

### 4.5 Database of *Iris* Secondary Metabolites

To perform the UHPLC-HRMS/MS targeted screening of phytochemicals in *Iris* extracts, an in-house database of secondary metabolites found in various *Iris* species was compiled based on scientific literature.^4,8–16^ The database includes 288 compounds, covering (iso)­flavonoids, terpenoids, steroids, stilbenes, quinones, phenols, phenolic acids, xanthones, and other unclassified compounds. For statistical and differential analysis, these compounds were further categorized into more specific compound groups (e.g., isoflavonoid glycosides, *C*-glycosyl xanthones, and flavanones). The preparation of the database was initiated within our previous study,^44^ and the final version is provided in Supporting Information, S2.

### 4.6 UHPLC-HRMS/MS Data Processing and Compound Identification

The targeted screening was carried out using the in-house database of secondary metabolites reported in various *Iris* species, as outlined in Section 4.5 Database of *Iris* Secondary Metabolites. Raw data were processed with Agilent MassHunter Profinder 8.0 software, generating a data matrix that included the tentative identification of the detected compounds based on MS data and the analytical signals across the analyzed samples. The extraction parameters for peak selection were as follows: minimal ion intensity ≥ 5000 counts; type of ions: [M + H]^+^, [M + NH_4_]^+^, [M – H]^−^, [M + HCOO]^−^; mass accuracy ≤ 5 ppm; *Q*-score ≥ 80%. Duplicate entries for compounds ionizing in both negative and positive polarities were removed based on the signal intensity and peak shape. Compounds producing an MS/MS spectrum of the (de)­protonated ion were further evaluated using the in silico fragmentation software Agilent MassHunter Molecular Structure Correlator 8.0 (MSC), and those with a match score with the theoretical MS/MS spectrum below 60% were excluded from the final list. Where possible, MS/MS spectra of the most significant compounds were compared to experimental MS/MS spectra from online spectral libraries obtained using comparable instrumentation and conditions: MassBank of North America (MoNA, mona.fiehnlab.ucdavis.edu), MassBank (massbank.eu), Human Metabolome Database (HMDB; hmdb.ca), or the scientific literature.

Given the lack of online available experimental MS/MS spectra or reference standards for many plant metabolites, most detected compounds were tentatively identified at levels 2 and 3, according to a recent review.^78^ The identification confidence was strengthened by the fact that all of the targeted metabolites had been previously identified in the studied plant material. In many cases, due to the similarity of MS/MS spectra among certain isomers, it was not possible to definitively assign a single structure.

### 4.7 Chemometric Analysis

Chemometric analysis of the UHPLC-HRMS/MS data was conducted using the MetaboAnalyst (https://www.metaboanalyst.ca/) and SIMCA 17.0 (Sartorius) tools. Data pretreatment included replacement of missing values with one-fifth of the lowest detected signal, followed by total area sum normalization applied to the complete data set. For each chemometric analysis, the normalized data were then subjected separately to log transformation and Pareto scaling to approximate normal distribution and ensure balanced variance across features. The preprocessed data were then used to generate an unsupervised Principal Component Analysis (PCA) model.

Insignificant features, which did not contribute to the differentiation between sample groups, were filtered using a Volcano plot for each experimental factor separately (subgenus, plant part, cultivation method, and plant treatment). The Fold Change threshold was set at 2.0, and the p-value threshold was set at 0.1 by default. Reducing the number of features (compounds) helped prevent overfitting and enhance the reliability of subsequent supervised Orthogonal Partial Least Squares Discriminant Analysis (OPLS-DA) models. The quality of the statistical models was assessed using the interpretation parameters R^2^X and R^2^Y, and the predictive ability parameter *Q* ^2^, validated by 7-round internal cross-validation. A threshold of Variable Importance in Projection (VIP) score of >1 was set for significant differential metabolites.

To identify potential bioactive compounds, correlational analysis based on the Pearson correlation coefficient (*r*) was applied on the combined bioassay and sum-normalized phytochemical profile data, as done in other studies.^79–81^ Compounds with *r* ≥ 0.4 were considered moderately correlated, *r* ≥ 0.6 was considered highly correlated, and *r* ≥ 0.8 was considered very highly correlated. The significance value was set at *p* = 0.01.

## Supplementary Material



## Notes

Overview of the analyzed *Iris* samples, in-house database of secondary metabolites of *Iris* spp., overview of metabolites detected in the experimental *Iris* sample set using UHPLC-HRMS/MS, volcano plot list of significantly up- and downregulated metabolites in the subgenus *Iris* and *Limniris*, volcano plot list of significantly up- and downregulated compound groups in the subgenus *Iris* and *Limniris*, volcano plot list of significantly up- and downregulated metabolites in the leaves and roots with rhizomes of *Iris* spp., volcano plot list of significantly up- and downregulated metabolites in aeroponics and hydroponics , and phytochemical profiles and bioactivity assay data for the experimental *Iris* sample set (XLSX)

## References

[1] Crişan I., Cantor M. (2017). New perspectives on medicinal properties and uses of
Iris sp. Hop Med. Plants.

[2] Duka I., Maleš Ž., Bojić M., Hruševar D., Mitić B. (2020). Chemical Fingerprinting, Total Phenolics
and Antioxidant
Activity of Some Iris Taxa. Croat. Chem. Acta.

[3] Okba M. M., Abdel Baki P. M., Khaleel A. E., El-Sherei M. M., Salem M. A. (2021). Discrimination of common Iris species from Egypt based
on their genetic and metabolic profiling. Phytochem.
Anal..

[4] Yehia S. M., Ayoub I. M., Watanabe M., Devkota H. P., Singab A. N. B. (2023). Metabolic
profiling, antioxidant, and enzyme inhibition potential of Iris pseudacorus
L. from Egypt and Japan: A comparative study. Sci. Rep..

[5] Kim J.-L. (2012). Osteogenic Activity
of Yellow Flag Iris (Iris pseudacorus) Extract
Modulating Differentiation of Osteoblasts and Osteoclasts. Am. J. Chin. Med..

[6] Michalak A. (2021). Iris pseudacorus as an easily accessible source of antibacterial
and cytotoxic compounds. J. Pharm. Biomed. Anal..

[7] Caspers, Z. ; Sekerka, P. Zahradní Kosatce a Jejich Šlechtění v České Republice; Středisko společných činností AV ČR: Prague, 2019.

[8] Kaššák P. (2013). Total flavonoids
and phenolics content of the chosen genus Iris species. Acta Univ. Agric. Silvic. Mendelianae Brunensis.

[9] Kaššák P. (2013). Secondary
metabolites of the choosen Genus Iris species. Acta Univ. Agric. Silvic. Mendelianae Brunensis.

[10] Alperth F. (2019). Metabolic profiling of rhizomes of native populations
of the strictly
endemic Croatian species Iris adriatica. Plant
Biosyst..

[11] Kukula-Koch W. (2015). Major secondary metabolites
of Iris spp. Phytochem.
Rev..

[12] Kostić A. Ž., Gašić U. M., Pešić M. B., Stanojević S. P., Barać M. B., Mačukanović-Jocić M. P., Avramov S. N., Tešić Ž.
L. (2019). Phytochemical Analysis
and Total Antioxidant Capacity of Rhizome, Above-Ground Vegetative
Parts and Flower of Three Iris Species. Chem.
Biodivers.

[13] Mykchailenko O. O., Kovalyov V. M. (2016). Phenolic compounds
of the genus Iris plants (Iridaceae). Czech
and Slovak Pharmacy.

[14] Bukvički D., Novaković M., Ab Ghani N., Marin P. D., Asakawa Y. (2018). Secondary
metabolites from endemic species Iris adriatica Trinajstić
ex Mitić (Iridaceae). Nat. Prod. Res..

[15] Roger B., Jeannot V., Fernandez X., Cerantola S., Chahboun J. (2012). Characterisation and Quantification
of Flavonoids in
Iris germanica L. and Iris pallida Lam. Resinoids from Morocco. Phytochem. Anal..

[16] Amin H. I. M., Hussain F. H. S., Najmaldin S. K., Thu Z. M., Ibrahim M. F., Gilardoni G., Vidari G. (2021). Phytochemistry and Biological Activities of Iris Species
Growing in Iraqi Kurdistan and Phenolic Constituents of the Traditional
Plant Iris postii. Molecules.

[17] Mykhailenko O. (2020). Qualitative and Quantitative Analysis of Ukrainian Iris Species:
A Fresh Look on Their Antioxidant Content and Biological Activities. Molecules.

[18] Azaizeh H. (2012). Effects of
Mineral Nutrients on Physiological and Biochemical Processes Related
to Secondary Metabolites Production in Medicinal Herbs. Med. Aromat. Plant Sci. Biotechnol..

[19] Mykhailenko O. (2020). Effect of ecological factors on the accumulation
of phenolic compounds
in Iris species from Latvia, Lithuania and Ukraine. Phytochem. Anal..

[20] Atherton H. R., Li P. (2023). Hydroponic Cultivation
of Medicinal Plants—Plant Organs and
Hydroponic Systems: Techniques and Trends. Horticulturae.

[21] Sharma U., Kataria V., Shekhawat N. S. (2018). Aeroponics
for adventitious rhizogenesis
in evergreen haloxeric tree Tamarix aphylla (L.) Karst.: influence
of exogenous auxins and cutting type. Physiol.
Mol. Biol. Plants.

[22] Chaturvedi V., Agrawal K., Verma P. (2021). Chicken feathers:
a treasure cove
of useful metabolites and value-added products. Environ. Sustain..

[23] Surendran U., Chandran C., Joseph E. J. (2016). Hydroponic
cultivation of Mentha
spicata and comparison of biochemical and antioxidant activities with
soil-grown plants. Acta Physiol. Plant..

[24] Souret F. F., Weathers P. J. (2000). The growth of saffron (Crocus sativus
L.) in aeroponics
and hydroponics. J. Herbs, Spices, Med. Plants.

[25] Selwal N. (2023). Enhancing secondary metabolite production in plants:
Exploring traditional
and modern strategies. J. Agric Food Res..

[26] Zhao Y., Cartabia A., Lalaymia I., Declerck S. (2022). Arbuscular
mycorrhizal
fungi and production of secondary metabolites in medicinal plants. Mycorrhiza.

[27] Porcel R., Aroca R., Ruiz-Lozano J. M. (2012). Salinity
stress alleviation using
arbuscular mycorrhizal fungi. A review. Agron.
Sustain. Dev..

[28] Xing S. (2024). Arbuscular mycorrhizal
symbiosis alleviates arsenic phytotoxicity
in flooded Iris tectorum Maxim. dependent on arsenic exposure levels. Environ. Pollut..

[29] Zhao W. (2023). Metagenomics reveal
arbuscular mycorrhizal fungi altering functional
gene expression of rhizosphere microbial community to enhance Iris
tectorum’s resistance to Cr stress. Sci.
Total Environ..

[30] Crişan I., Vidican R., Olar L., Stoian V., Morea A., Ştefan R. (2019). Screening
for Changes on Iris germanica L. Rhizomes
Following Inoculation with Arbuscular Mycorrhiza Using Fourier Transform
Infrared Spectroscopy. Agronomy.

[31] Bhat G. (2014). HPLC-DAD-ESI-MS/MS
Identification and Characterization of Major Constituents of Iris
crocea, Iris germanica and Iris spuria Growing in Kashmir Himalayas,
India. J. Anal. Bioanal. Tech..

[32] Baser K. H. C. (2011). Composition of Volatiles from Three Iris Species
of
Turkey. J. Essent. Oil Res..

[33] Mykhailenko O., Kovalyov V., Orlova T. (2020). Chemical composition
of the essential
oil of several Iris species. Thai J. Pharmaceut.
Sci..

[34] Mykhailenko O. (2018). Composition
of Volatile Oil of Iris pallida Lam. from Ukraine. Turk. J. Pharm. Sci..

[35] Moein M. R. (2008). Flavonoids from Iris
songarica and their antioxidant and estrogenic
activity. Planta Med..

[36] Wei Y., Shu P., Hong J., Qin M. (2012). Qualitative and Quantitative Evaluation
of Phenolic Compounds in Iris dichotoma Pall. Phytochem. Anal..

[37] Wollenweber E. (2003). Cancer chemopreventive
in vitro activities of isoflavones isolated
from Iris germanica. Planta Med..

[38] Fang R., Houghton P. J., Hylands P. J. (2008). Cytotoxic effects of compounds from
Iris tectorum on human cancer cell lines. J.
Ethnopharmacol..

[39] Hacibekiroǧlu I., Kolak U. (2011). Antioxidant
and anticholinesterase constituents from the petroleum
ether and chloroform extracts of Iris suaveolens. Phytother. Res..

[40] Shu P., Hong J.-L., Wu G., Yu B.-Y., Qin M.-J. (2010). Analysis
of Flavonoids and Phenolic Acids in Iris tectorum by HPLC-DAD-ESI-MSn. Chin J. Nat. Med..

[41] Mykhailenko O., Kovalyov V., Kovalyov S., Krechun A. (2017). Isoflavonoids from
the rhizomes of Iris hungarica and antibacterial activity of the dry
rhizomes extract. Ars Pharm..

[42] Mocan A. (2018). Biological effects and chemical characterization of Iris schachtii
Markgr. extracts: A new source of bioactive constituents. Food Chem. Toxicol..

[43] Zhu B. (2024). Knowledge-based in silico
fragmentation and annotation of mass spectra
for natural products with MassKG. Comput. Struct.
Biotechnol. J..

[44] Jaegerova T. (2024). Investigation of Iris
versicolor metabolic profile and optimization
of the isolation of bioactive components on a semi-operation scale. Process Biochem..

[45] Wilson C. (2006). Patterns in
Evolution in Characters That Define Iris Subgenera and Sections. Aliso.

[46] Wang H., Cui Y., Zhao C. (2010). Flavonoids
of the Genus Iris (Iridaceae). Mini-Rev. Med.
Chem..

[47] Li L., Chen Y., Feng X., Yin J., Li S., Sun Y., Zhang L. (2019). Identification of Metabolites
of Eupatorin in Vivo
and in Vitro Based on UHPLC-Q-TOF-MS/MS. Molecules.

[48] Qin M.-J., Xu L.-S., Toshihiro T., Wang Q., Xu G.-J. (2000). A preliminary
study on the distribution pattern of isoflavones in rhizomes of Iris
from China and its systematic significance. J. Syst. Evol..

[49] Yu J., Lee J.-H., Song M.-H., Keum Y.-S. (2023). Metabolomic Responses
of Lettuce (Lactuca sativa) to Allelopathic Benzoquinones from Iris
sanguinea Seeds. J. Agric. Food Chem..

[50] Williams C. A., Harborne J. B., Colasante M. (1997). Flavonoid
and xanthone patterns in
bearded Iris species and the pathway of chemical evolution in the
genus. Biochem. Syst. Ecol..

[51] Zhang Y.-Y., Wang Q., Qi L.-W., Qin X.-Y., Qin M.-J. (2011). Characterization
and determination of the major constituents in Belamcandae Rhizoma
by HPLC–DAD–ESI-MSn. J. Pharm.
Biomed. Anal..

[52] Amani
Machiani M., Javanmard A., Habibi Machiani R., Sadeghpour A. (2022). Arbuscular mycorrhizal Fungi and Changes in Primary
and Secondary Metabolites. Plants.

[53] Schmid C. (2021). Quantitative Mapping of Flavor and Pharmacologically Active Compounds
in European Licorice Roots (Glycyrrhiza glabra L.) in Response to
Growth Conditions and Arbuscular Mycorrhiza Symbiosis. J. Agric. Food Chem..

[54] Araim G., Saleem A., Arnason J. T., Charest C. (2009). Root Colonization by
an Arbuscular Mycorrhizal (AM) Fungus Increases Growth and Secondary
Metabolism of Purple Coneflower, Echinacea purpurea (L.) Moench. J. Agric. Food Chem..

[55] Zhao Y., Cartabia A., Lalaymia I., Declerck S. (2022). Arbuscular mycorrhizal
fungi and production of secondary metabolites in medicinal plants. Mycorrhiza.

[56] Walid N., Kassam R., Ashfaq M. (2021). Interaction
between mycorrhizal and
medicinal plants towards enhancement of secondary metabolites. Int. J. Chem. Stud..

[57] Crişan I., Vidican R., Olar L., Stoian V., Morea A., Ştefan R. (2019). Screening
for Changes on Iris germanica L. Rhizomes
Following Inoculation with Arbuscular Mycorrhiza Using Fourier Transform
Infrared Spectroscopy. Agronomy.

[58] Ran Z. (2022). Arbuscular mycorrhizal fungi: Effects on secondary metabolite accumulation
of traditional Chinese medicines. Plant Biol..

[59] Selwal N. (2023). Enhancing secondary metabolite production in plants: Exploring traditional
and modern strategies. J. Agric Food Res..

[60] Hu T. (2021). Metabolite identification of iridin in rats by using
UHPLC-MS/MS
and pharmacokinetic study of its metabolite irigenin. J. Chromatogr. B.

[61] BioCat. Iriflophenone 2-O-Rhamnoside. https://www.biocat.com/products/TN3870-5mg-TM (accessed 4.2.2025).

[62] Wahyuningsih M. (2005). Phalerin,
A New Benzophenoic glucoside isolated from the methanol
extract of Mahkota Dewa (Phaleria macrocarpa (Scheff). Boerl. Leaves. Indones. J. Pharm..

[63] Bhosale P.-B., Vetrivel P., Ha S. E., Kim H. H., Heo J. D., Won C. K., Kim S. M., Kim G. S. (2021). Iridin Induces G2/M
Phase Cell Cycle Arrest and Extrinsic Apoptotic Cell Death through
PI3K/AKT Signaling Pathway in AGS Gastric Cancer Cells. Molecules.

[64] Pei K., Ou J., Huang J., Ou S. (2016). Coumaric acid and its conjugates:
dietary sources, pharmacokinetic properties and biological activities. J. Sci. Food Agric..

[65] Hu J. (2023). p-coumaric acid prevents
Colletotrichum gloeosporioides by inhibiting
membrane targeting and organic acid metabolism. Postharvest Biol. Technol..

[66] Xu J.-G., Hu H. X., Chen J. Y., Xue Y. S., Kodirkhonov B., Han B. Z. (2022). Comparative study
on inhibitory effects of ferulic
acid and p-coumaric acid on Salmonella Enteritidis biofilm formation. World J. Microbiol. Biotechnol..

[67] Jaudszus A. (2012). Vaccenic acid-mediated reduction in cytokine production is independent
of c9,t11-CLA in human peripheral blood mononuclear cells. Biochim. Biophys. Acta, Mol. Cell Biol. Lipids.

[68] Jacome-Sosa M. (2016). Vaccenic acid suppresses
intestinal inflammation by increasing anandamide
and related N-acylethanolamines in the JCR:LA-cp rat­[S]. J. Lipid Res..

[69] Mostafaie A., Mansouri K., Abbasi A., Bahrami G., Sisakhtnejad S. (2015). The effect
of Cis and trans vaccenic acids on expression of ICAM-1 and VCAM-1
in human microvascular endothelial cells (HMEC). J. Rep. Pharm. Sci..

[70] Vaou N., Stavropoulou E., Voidarou C., Tsigalou C., Bezirtzoglou E. (2021). Towards Advances
in Medicinal Plant Antimicrobial Activity: A Review Study on Challenges
and Future Perspectives. Microorganisms.

[71] Wagner H., Ulrich-Merzenich G. (2009). Synergy research: Approaching a new
generation of phytopharmaceuticals. Phytomedicine.

[72] Deprez, M. ; Robinson, E. C. Chapter 8 - Feature extraction and selection. in Machine Learning for Biomedical Applications, Deprez, M. , Robinson, E. C. , Eds., pp 175–192, Academic Press, 2024. doi:DOI: 10.1016/B978-0-12-822904-0.00013-3.

[73] Turk A., Addam K., Hwang B. Y., Lee M. K. (2025). Metabolomic Profiling
of Iris palaestina via Molecular Networking and Its Anti-Diabetic
Potential. Molecules.

[74] Zuzana, C. ; Botanical Gardens As A Part Of European Cultural Heritage Iris, 2020.

[75] Szemerédi N., Dobiasová S., Salardón-Jiménez N., Kincses A., Nové M., Habibullah G., Sevilla-Hernández C., Benito-Lama M., Alonso-Martínez F. J., Viktorová J. (2021). Cyano- and Ketone-Containing Selenoesters as Multi-Target Compounds
against Resistant Cancers. Cancers.

[76] Viktorová J., Dobiasová S., Řehořová K., Biedermann D., Káňová K., Šeborová K., Václavíková R., Valentová K., Ruml T., Křen V. (2019). Antioxidant, Anti-Inflammatory, and Multidrug Resistance Modulation
Activity of Silychristin Derivatives. Antioxidants.

[77] Tran V. N. (2023). Cannabidiol nanoemulsion for eye treatment – Anti-inflammatory,
wound healing activity and its bioavailability using in vitro human
corneal substitute. Int. J. Pharm..

[78] Schymanski E. L. (2014). Identifying Small Molecules via High Resolution
Mass Spectrometry:
Communicating Confidence. Environ. Sci. Technol..

[79] Ghosh, P. , Das, C. , Biswas, S. , Nag, S. K. , Dutta, A. , Biswas, M. , Sil, S. , Hazra, L. , Ghosh, C. , Das, S. , , Phytochemical composition analysis and evaluation of in vitro medicinal properties and cytotoxicity of five wild weeds: A comparative study. (2020).9, 493, 10.12688/f1000research.22966.1, PMC7331102 32676186

[80] Zhang P., Wang H., Xu X., Ye Y., Zhang Y. (2024). Correlation
analysis between phytochemical composition and biological activities
of Artemisia scoparia. Food Biosci..

[81] Koss-Mikołajczyk I., Kusznierewicz B., Bartoszek A. (2019). The Relationship between Phytochemical
Composition and Biological Activities of Differently Pigmented Varieties
of Berry Fruits; Comparison between Embedded in Food Matrix and Isolated
Anthocyanins. Foods.

